# Wisconsin State Medical Society

**Published:** 1870-09

**Authors:** 


### WISCONSIN STATE MEDICAL SOCIETY.

The Medical Society of the State of Wisconsin held its regular annual session in Milwaukee, on the 15th, 16th, and 17th days of June, its President, Dr. S. Marks, of Milwaukee, presiding.

After an opening prayer, by the Rev. G. M. Stone, the Society was addressed by Dr. J. K. Bartlett, Chairman of the Committee of Arrangements, who welcomed it to this city, in which it has not before held a session for twenty years.

Pending the report of the Board of Censors, in reference to admission of new members, a discussion was opened by Dr. Carley, who reported a case of what he thought to be apoplexy, following delivery, in which bleeding and purgatives were heroically administered, and recovery rapid.

Dr. Van Norstrand inquired if paralysis followed in the case, and receiving a negative reply, said that, in his opinion, the case was one of puerpural convusions, as did also Drs. Meacher, Meachum, and Brewster, who participated in the discussion, sustaining the treatment of Dr. Carley, though differing from him in the diagnosis.

The Censors made report recommending for membership the names of a number of physicians examined by them, and upon this and subsequent reports of this Board, there were received into the Society forty-nine new-members.

Reports of Committees being called for, Dr. Mason, from the Committee on Surgery, read an interesting essay on the uses of Carbolic Acid.

Dr. Meacher, from the same Committee, read an essay on Colles's Fracture, advocating the use of an inter-osseous compress.

Dr. C. Linde presented an essay, being "An Historical Outline of Traumatic Surgery."

Dr. Davis read the history of a case of lithotomy in a child of 5 years, resulting in the death of the child in 40 hours, from peritonitis.

Dr. Armstrong read the history of a case of breech presentation at full time, in which the head of the child had actually been torn from the body by the attendants, the woman having previously had several living children. Two *doctors* had been in attendance, but had made no effort to deliver the woman with instruments, and had finally abandoned the case. The head alone was left in the pelvic cavity when the Doctor arrived, and by him was delivered with forceps and craniotomy. The woman died in 24 hours, apparently "from shock of the system alone."

Dr. Barrett read a paper detailing a case of obstetrics, in which a woman was delivered of an eight-month's still-born child, and in a few hours of another fœtus of the size of two and a-half or three months. The woman had met with a fall in the early period of pregnancy, to which the Doctor attributed the death of the smaller fœtus.

Dr. Mason reported a case, in which, after a natural labor, he was in a few hours recalled to the case, and found that a five-month's fœtus, with placenta attached, had been expelled, but "in a mummified condition."

Dr. Coon reported a case in which a living and healthy child was born, three months after the mother had been delivered of another child also healthy and living.

Dr. Palmer mentioned a case in which a seven-month's fœtus was found to be entirely destitute of any trace of osseous matter.

Brief histories of other anomalous cases were given by Drs. Carley, Davis, and others.

Dr. Ferris presented and read two papers, the first being the History of a Case of Enlarged Prostrate; and the second being an Essay on Trismus Nascentium, in which disease, the Doctor thinks "the proper pathological condition is undue pressure upon the medulla oblongata and the nerves originating from it, produced by displacement of the cranial bones, especially the occipital."

The proceedings of the Society, thus synoptically presented, occupied its attention during the sessions of Wednesday evening and Thursday morning, and in the afternoon of that day the Society, with invited guests, were, by the liberal provisions of the Committee of Arrangements, entertained with an excursion on Lake Michigan.

Upon assembling again in the evening, the Annual Address of the President was delivered, it being a review of the position occupied by the profession at the present time, as compared with its position in past ages, and urging, in view of our superior advantages, correspondingly higher attainments—claiming for Medical Societies an exalted position in this work, and for our own Society, urging it to be "not only our duty to see that applicants for admission have the required qualifications at the time of admission, but that they continue to keep pace with the advancement of the profession," and that "if we would triumph over quackery we should elevate ourselves so much above the mere pretender in medicine, that the most casual observer cannot fail to mark well the distinction between us."

Dr. Witter introduced a resolution looking to the establishment of a Medical Department of the State University, which, after discussion, was laid on the table.

Dr. Cody, from the Committee on New Remedies, read an essay on the Hydrate of Chloral, praising it as a sedative and hypnotic, and as a safe and valuable medicine in all cases where an anodyne is required—ranks it next to opium, and as, in many cases, preferable to that drug—has found it "especially beneficial in a large class of nervous and delicate females, afflicted with the class of diseases known as neuroses—has given from five to fifteen grains as an anodyne, and from thirty to sixty grains as a hypnotic—has not in any case seen unpleasant effects from its use. The paper elicited considerable discussion, exhibiting a variety of experience with the drug, mainly corroborative of that of Dr. Cody.

Dr. Palmer, having taken the medicine, found it to relieve pain and produce sleep, but the sleep was of a disturbed character, and was followed by great languor and by irritability of the stomach; had obtained no satisfactory results from its use.

Dr. Page's experience was similar to that of Dr. Palmer.

Dr. Cody also read a paper prepared by Dr. Cory, giving the history of a case of pleuro-pneumonia, in which there had been expectorated a perfect fibrinous cast of a bronchial tube (which was presented with the paper), following which recovery was rapid.

Dr. Cory presented another paper, giving the history of a case of insanity, treated with bromide of potassium with good results.

Dr. Waterhouse presented a paper on the "Management of Disease," the special point of which was the position of patients with reference to the law of gravitation—"an appeal for better management and less drugs."

Dr. Barrett gave the history of a case of hydrocele successfully treated by injection of chloroform and compound tincture of iodine.

Dr. Stoddard presented an essay on the "Pathology of Pulmonary Consumption," following Lebert "in denying the existence of specific tuberculosis," maintaining that "tubercles may be produced by ordinary irritants under certain conditions, and these conditions being understood will give us a fair field to combat their morbid tendencies."

Dr. Treat, Chairman of a Committee appointed at a previous meeting, to secure the passage of a law legalizing dissections, reported that the Committee had secured the passage of such a law, which may be found among the General Laws of the State, entitled "An Act to Legalize Dissection, Approved February 9th, 1868."

A communication was received from Dr. D. C. Davies, regretting his inability to attend, and suggesting the appointment of a Committee on Gynæcology, to report at the next meeting, which suggestion was adopted, and Dr. Davis was appointed on such Committee.

Society adjourned until Friday morning, when the first business in order was the election of officers for the ensuing year; the election resulted as follows:—

*President*—Dr. H. P. Strong, of Beloit.

*1st Vice-President*—Dr. Darius Mason, of Prairie du Chien.

*2nd Vice-President*—Dr. A. H. Van Norstrand, of Madison.

*Secretary*—Dr. J. T. Reeve, of Appleton.

*Corresponding Secretary*—Dr. R. D. McArthur, of Milwaukee.

*Treasurer*—Dr. J. T. Reeve, of Appleton.

Drs. J. K. Bartlett, H. Van Duzen, and L. J. Barrows were re-elected censors.

Dr. Van Duzen reported an extremely interesting case, in which, on making a *post mortem* examination, there was found no trace whatever of the left lung, its cavity being occupied by a number of small, irregularly-shaped pieces of bone, which were exhibited. The other lung was healthy, and there was no trace of disease elsewhere.

Dr. Dalton, who also knew of the case, stated that the man had been a sailor, and that many years before his death, he had sustained a fall, injuring that side.

Dr. Bartlett presented an essay on "Intra-Uterine Medication," which, in consideration of the lateness of the hour, was referred to the Committee on Publication, as was also a paper by Dr. Armstrong on the "Constitutionality of Disease," and one by Dr. Brewster on "Criminal Abortion."

Dr. Bartlett offered a resolution, which was adopted, empowering the Board of Censors to meet three days prior to the meeting of the Society, for the examination of applicants for membership.

Resolutions of thanks to the Officers of the Society, to the Committee of Arrangements, to the physicians and citizens of Milwaukee, and to the press, were then adopted.

Dr. Manley presented the history of a case of opium eating, in which persistence in the use of the drug had not produced a necessity for an increase of its dose, and in which a cure was effected by the use of quinine, and gradually diminishing doses of tr. opii.

Dr. Johnson read an essay on Torpid Bowels, condemning the common use of purgatives, and particularly commending the use of Graham bread.

The following named gentlemen were elected as delegates to the next meeting of the National Medical Association:

Drs. Waterhouse, Marks, H. Van Duzen, Wight, J. K. Bartlett, Reynolds, Strong, Reeve, D. C. Davies, Whiting, Wolcott, Treat, J. Johnson, Van Norstrand, Mason, Russell, Favelli, Burroughs, Fuller, and Dickson.

The Standing Committees for the ensuing year were announced as follows:

*Arrangements*—Drs. Marks, Johnson, and Fuller.

*Surgery*—Drs. Marks, Mason, and Dalton.

*Practice*—Drs. Whiting, Page, and Manley.

*Obstetrics*—Drs. J. K. Bartlett, Ferris, and Armstrong.

*Pathology*—Drs. Van Norstrand, Thorndike, and Stoddard.

*New Remedies*—Drs. Faville, Russell, and Waterhouse.

*Medical Education*—Drs. Wight and Whiting.

*Diseases of Eye*—Dr. E. W. Bartlett.

On motion, the Secretary was directed to furnish a synopsis of the proceedings of this Convention for publication in the *Medical and Surgical Reporter*, Philadelphia, and in the *Chicago Medical Examiner.*

Society adjourned to meet in Milwaukee on the third Wednesday of June, A.D. 1871. J. T. R.

